# Localized malignant mesothelioma in the stomach and mediastinum

**DOI:** 10.1093/icvts/ivab276

**Published:** 2021-09-29

**Authors:** Tanita Drejer Jeppesen, Anette Højsgaard, Daniel Kjær, Thomas Decker Christensen

**Affiliations:** 1 Department of Cardiothoracic and Vascular Surgery, Aarhus University Hospital, Aarhus, Denmark; 2 Department of Surgery, Aarhus University Hospital, Aarhus, Denmark; 3 Department of Clinical Medicine, Aarhus University, Aarhus, Denmark

**Keywords:** Localized malignant mesothelioma, Mediastinum, Stomach

## Abstract

Localized malignant mesothelioma is rare. It has a histological pattern identical to diffuse malignant mesothelioma but without diffuse serosal spread. Localized malignant mesothelioma typically originates from the pleura, peritoneum or pericardium, but can occasionally develop from organs. Our cases represent what might be the largest mediastinal localized malignant mesothelioma described and the first presentation of the epithelioid type in the stomach of an adult.

## INTRODUCTION

Localized malignant mesothelioma (LMM) is a rare neoplasm [1]. The histological pattern is identical to diffuse malignant mesothelioma (DMM) but without diffuse serosal spread [1]. LMM typically originates from the pleura, peritoneum, pericardium or tunica vaginalis [1, 2], but can occasionally develop in organs, such as lungs, gonads, liver, spleen, pancreas and the ventricle [1]. We present 2 cases with very uncommon presentations of LMM. To our knowledge, our cases represent the largest mediastinal LMM described and the first presentation of epithelioid LMM in the stomach of an adult.

## CASE DESCRIPTION

### Case 1

A 66-year-old male, previously asbestos-exposed, non-smoker, presented with 3 months of intermittent fever, fatigue, mild cough, right-sided chest pain and a weight loss of 7 kg.

**Figure 1:**
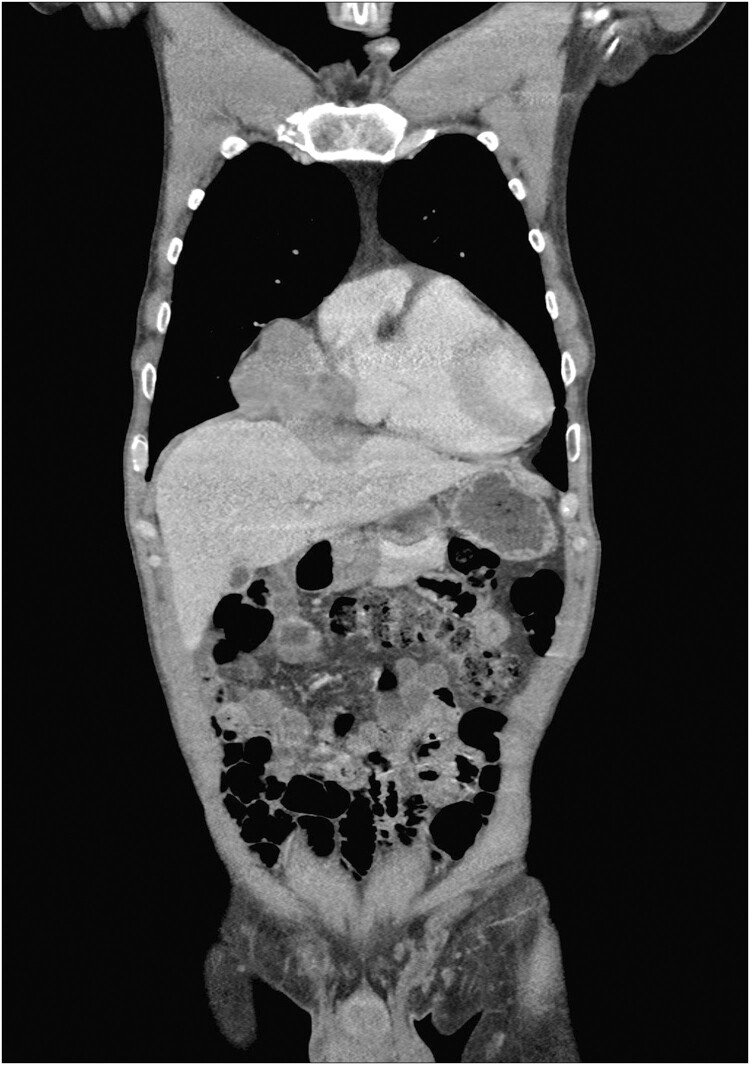
Computed tomography scan showed an 8 × 6 × 9 cm tumorous process along the right side of the heart.

A positron emission tomography-computed tomography (CT) showed an 8 × 6 × 9 cm tumour along the right side of the heart (Fig. 1). A biopsy revealed adenocarcinoma.

The tumour was considered a primary lung cancer, adjacent to the mediastinum and diaphragm and thought to be primary resectable. A right-sided anterior muscle-sparing thoracotomy was performed with the intention of resecting the tumour.

During exploration, the tumour was found to extend more widely than expected. The thoracic part of the tumour was located on the right side of the mediastinum, measured 10 × 6 × 6 cm and continued in a large intraabdominal component, with no involvement of the right lung. The tumour was adjacent to the pericardium and right diaphragm. Re-evaluation of the preoperative CT and positron emission tomography-CT by liver surgeon and radiologist also suggested invasion to the left liver lobe, resembling an upper gastrointestinal tumour, which perioperative biopsy could not rule out. A perioperative gastroscopy showed no involvement of the oesophagus.

The procedure was terminated in order to re-evaluate and plan. The scans were consulted with a liver surgeon, and the surgical access was planned differently.

Three weeks later, definitive surgery was performed: In toto resection of the tumour through a laparoscopic and midline trans-sternal approach. By laparoscopy, the tumour was dissected from the liver. There were no signs of invasion into the liver nor diaphragm from the abdomen. From the sternotomy, the mediastinal tumour including the affected pericardium and diaphragm was resected. For reconstruction of pericardium and diaphragm, 2 Goretex patches were used.

Histological analysis revealed malignant epithelioid mesothelioma assumed to derive from the mediastinum.

The diaphragmatic resection margins contained tumour cells and the patient underwent adjuvant chemotherapy. A regime of 6 cycles of Cisplatin/Pemetrexed was planned, but only 3 cycles were given due to neutropenia.

A CT scan 6 months after surgery detected recurrent disease with an 8-mm pleuritic thickening resulting in additional chemotherapy and subsequent palliative radiotherapy due to tumour progression and symptoms of dyspnoea and thoracic pain. After the conclusion of palliative radiotherapy, 22 months after surgery, the symptoms improved. The patient has resumed his usual daily activities, e.g. able to take a bicycle ride of 30 km. CT scans show stable cancer disease over a month. The patient is now part of a project of experimental oncological treatment.

### Case 2

**Figure 2:**
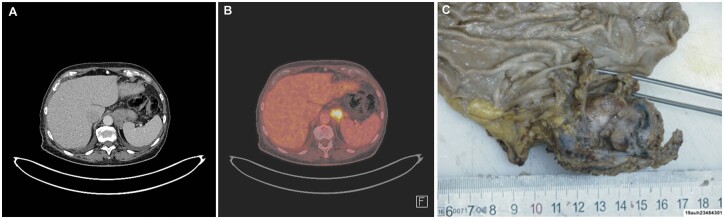
Computed tomography scan (A) and positron emission tomography-computed tomography scan (B) showed a 5-cm tumour located in close relation to the fundus of the stomach. (C) Photo of the resected material in Case 2.

A 74-year-old female, former smoker, presented with long-lasting abdominal pain, and a weight loss of 5–7 kg. CT scan (Fig. 2A) and positron emission tomography-CT (Fig. 2B) showed a 5-cm tumour located in close relation to the fundus of the stomach and left adrenal gland.

Gastroscopy and endoscopic ultrasound revealed an indentation in the gastric wall of the fundic area and a tumour with a diameter of 5 cm. Endoscopic ultrasound biopsy showed malignant mesothelioma, epithelioid type.

Systemic neoadjuvant chemotherapy was initiated. The patient received 6 cycles of Carboplatin/Pemetrexed resulting in a 1-cm tumour regression. Adjuvant radiotherapy was not included in the treatment strategy, partially because of problematic field delimitation.

A tumour adherent to the greater curvature of the stomach, diaphragm, aorta, spleen and pancreas was removed.

A laparoscopic approach was used. The tumour was dissected and freed from the close adherence to the aorta and the spleen, the relation to the pancreas warranted a tangential superficial wedge resection of the body and tail of the pancreas and then closure of the pancreatic defect with absorbable self-locking thread. In order to free the tumour, a wedge resection of the involved part of the diaphragm was performed. Finally, a proximal gastrectomy dividing the oesophagus just above the cardia and the stomach on the proximal part of the corpus was performed. Reconstructing continuity of the gastrointestinal tract was done with an oesophagogastric anastomosis using a circular OrVil 25-mm stapler device. The diaphragm was closed with several single stitches and covered with a BioMesh.

Postoperative histological results confirmed malignant mesothelioma of epithelioid type with clear resection margins.

The patient remains recurrence-free 22 months after surgery.

## DISCUSSION

LMM is rare with merely 101 cases described [1].

LMM is a serosal or subserosal, non-organ-specific tumour with a histological and immunohistochemically pattern identical to DMM, but without diffuse serosal spread [1, 3]. Approximately 90% of LMM originates from the pleura, but it can also derive from the peritoneum, pericardium and tunica vaginalis [1, 2]. Primary LMM can occasionally develop from organs, such as lungs, spleen, pancreas and the stomach [1].

When occurring in mediastinum, the mesothelial lining cells of the pericardium are considered as the most probable cells of origin [4].

LMM is often unexpected due to its rarity and clinical presentation, but it can occur in all ages, with males representing 70–75% of cases [1]. Asbestosis is a known risk factor of DMM [1, 4], but it is undetermined whether exposure to asbestos increases the risk of developing LMM [1].

Distinguishing mesothelioma from lung adenocarcinoma is histologically challenging when using only limited tissue from core biopsies [3]. The biopsies in our first case were mistaken for adenocarcinoma, and the diagnosis was unclear until the final histological analysis.

Early and correct diagnosis between LMM and DMM is important [1, 4], since DMM is a very aggressive neoplasm with a median survival of 6–18 months [1]. Distinguishing between LMM and DMM is also important, as the prognosis and the treatment options are different [1]. An estimated median survival for LMM is 29 months and for the epithelioid subtype 134 months [1, 5]. The difference in survival rates for DMM and LMM may be due to the fact that LMM often can be completely resected [5]. However, as seen in our first case, localized recurrence and even progression to DMM has been described after complete resection of LMM [5].

We have presented 2 unique cases: what might be the largest LMM tumour of mediastinal origin, with a rare tumour extension and disease recurrence, and the first epithelioid LMM with origin in the stomach in an adult.

These cases highlight the importance of considering mesothelioma in the differential diagnosis of a solid tumour, regardless of gender, asbestos exposure and especially organ of origin.

##  

**Conflict of interest:** Thomas Decker Christensen has been on the speaker bureaus for AstraZeneca, Boehringer-Ingelheim, Pfizer, Roche Diagnostics, Takeda, Merck Sharp & Dohme (MSD) and Bristol-Myers Squibb and has been in an Advisory Board for Bayer and MSD. O ther authors declared no conflict of interest.
